# The prospects for sustaining evidence-based responses to the US opioid epidemic: state leadership perspectives

**DOI:** 10.1186/s13011-020-00326-x

**Published:** 2020-11-04

**Authors:** Lauren Caton, Mina Yuan, Dexter Louie, Carlos Gallo, Karen Abram, Lawrence Palinkas, C. Hendricks Brown, Mark McGovern

**Affiliations:** 1 Center for Behavioral Health Services and Implementation Research, Department of Psychiatry and Behavioral Sciences, Stanford University School of Medicine, Palo Alto, CA USA; 2 Feinberg School of Medicine, Northwestern University, Chicago, IL USA; 3 Department of Children, Youth and Families, Dworak-Peck School of Social Work, University of Southern California, Los Angeles, CA USA; 4 Division of Primary Care and Population Health, Department of Medicine, Stanford University School of Medicine, Palo Alto, CA USA

**Keywords:** Sustainment, Sustainability, Opioid use disorder treatment, Opioid use disorder, Barriers and facilitators, Health policy, Grant funding

## Abstract

**Background:**

The US 21st Century Cures Act provided $7.5 billion in grant funding to states and territories for evidence-based responses to the opioid epidemic. Currently, little is known about optimal strategies for sustaining these programs beyond this start-up funding.

**Methods:**

Using an inductive, conventional content analysis, we conducted key informant interviews with former and current state leaders (*n* = 16) about barriers/facilitators to sustainment and strategies for sustaining time-limited grants.

**Results:**

Financing and reimbursement, service integration, and workforce capacity were the most cited barriers to sustainment. Status in state government structure, public support, and spending flexibility were noted as key facilitators. Effective levers to increase chances for sustainment included strong partnerships with other state agencies, workforce and credentialing changes, and marshalling advocacy through public awareness campaigns.

**Conclusions:**

Understanding the strategies that leaders have successfully used to sustain programs in the past can inform how to continue future time-limited, grant-funded initiatives.

## Background

Opioid overdose death is a major public health concern [1]. In response, the twenty-first Century Cures Act provided $7.5 billion in start-up funding to 57 states and territories administered through the Substance Abuse and Mental Health Services Administration (SAMHSA) [2]. This bolus in funding aimed to reduce overdose deaths through expansion of evidence-based treatment; including the FDA-approved medications of methadone, buprenorphine, and naltrexone. States employed a variety of evidence-based programs with this funding, ranging from peer navigators to post-overdose emergency department connection programs [3]. With this record funding comes a growing concern about the continuation, or sustainment [4], of these grant-financed programs [5]. This anxiety is reflected in public health service contexts, where scant research examines sustainment of evidence-based programs at the systems level [6–12]. Given the recent FY 2021 renewal of these grants, it is both timely and imperative to understand how policymaking state leaders attempt to ensure sustainment of these, and similar, programs.

Optimal strategies for sustainment are presently unknown. Emerging advancements in implementation science – the study of integrating evidence-based interventions into practice [4] – have developed sustainability definitions and frameworks [13–19]. One implementation science heuristic that conceptualizes sustainment is the four-phase Exploration, Preparation, Active Implementation, and Sustainment (EPIS) framework [9, 10]. The framework delineates an inner and outer context, referring to the intra-organizational and systems-wide levels respectively, within which factors involved in the four phases of implementation may operate [10]. Applications of EPIS focus on the first three phases within clinic and provider settings, not addressing the fourth phase - sustainment - or exploring its impact across systems-level contexts [20]. Additionally, pragmatic research in policy settings has yet to investigate factors that promote and undermine sustainment [7, 12, 21–23]. Beyond recent scientific support for tailoring strategies to identified barriers and facilitators, scant research investigates the applicability of tailoring for sustainment ventures [24–26]. Lack of research and tools leaves state leaders without support for increasing sustainment likelihood.

Documenting determinants (i.e. barriers and facilitators) that systems leaders face may aid in scientific understanding of sustainment in policy settings, and in developing potential strategies. To address this issue, we sought to:

1. delineate identified barriers and facilitators to sustainment of the opioid response or similar time-limited programs; and
2. suggest how strategies might be tailored to address typical barriers and facilitators to support sustainment.

Our method featured semi-structured interviews of a sample of current and former state leaders, with approximately equal representation by geography and tenure. We organized the discourse using the EPIS framework [10]. It is imperative we improve our capacity to sustain evidence-based programs in healthcare delivery systems that are launched by federal funding. The overarching goal of the present study is to describe key levers to sustainment of programs that have been implemented in response to the US opioid epidemic.

## Methods

This is a qualitative study featuring key informant interviews with eight former and eight current state agency leaders who oversee substance use prevention and treatment services within their respective states. This sample size, though small, represents the experience of 34% of all states across all leaders’ tenure (17 states; some leaders held the same role in multiple states). This also represents 16% of all possible individuals actively holding these positions. Interviews were oriented to ascertain perceptions on sustaining time-limited programs. All methods and reporting are in alignment with the consolidated criteria for reporting qualitative research (COREQ) guidelines [27].

### Participants

Leaders from the research team and a national organization of state agency directors identified and recommended state agency leaders with authority over federal grants for substance use treatment. In order to achieve qualitative saturation – adequate sample size for qualitative thematic determination [28] – eight former and eight current leaders were invited via email with intentional sampling diversity in US state, region, geographic size, and population density. Two leaders (one current and one former) denied initial invitations due to time constraints, so additional leaders were contacted for a final sample of 16 leaders. The leaders’ performance during their current or former tenure was not a criteria for inclusion in the study. Former leaders were not included for comparison, but to provide a tenured and historical perspective of individuals currently managing statewide programs. We did not match current or former leaders by state, and only 25% of the sample included leaders from the same state.

### Interview guides & process

The authors, skilled in qualitative research design, created two semi-structured interview guides, available upon request from the corresponding author. All systems leaders (participants) were asked to identify 1) examples of programs successfully sustained beyond the grant, 2) barriers and facilitators to continuing these programs, 3) strategies they employed in order to successfully sustain programs, and 4) recommendations for types of training or content they would find beneficial for continuing to sustain programs in the future. This study primarily reports on questions 2 and 3. Additionally, the current leaders were asked the same questions in regard to their work in sustaining programs within their purview, including but not limited to current opioid response-funded programs. Participants received a copy of the study abstract and IRB consent forms in advance, but not a copy of the interview guide unless requested. Interviews lasted approximately 1 h and were conducted by the male senior author. The interviewer holds a PhD in psychology, has expertise in clinical and mixed methods interviewing, and is a tenured professor at the parent institution. All interviews took place following introductions, explanation of purpose, and confidentiality agreements (aligned with exempt status from Stanford University Institutional Review Board). They were audio recorded via a secure videoconference platform where participant video was optional; the interviewer enabled video and audio. No repeat interviews were conducted. The interviewer took anonymized field notes during interviews and interviewee identities were blinded and de-identified for transcription and coding. Transcripts were not returned to participants for comment or correction, but transcriptions were verbatim with the exception of eliminated named identities.

### Data analysis

**Fig. 1:**
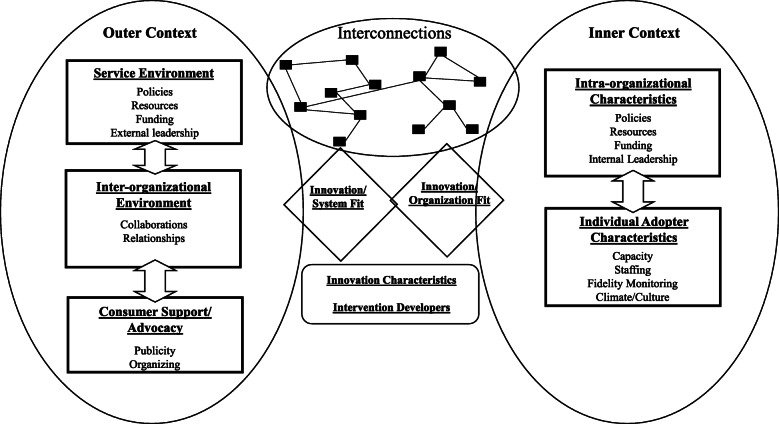
EPIS Framework with Inner and Outer Context Subdomains. ^a^“Individual” within the inner context of the EPIS framework is interpreted as unit-level factors influencing adoption of a practice within a system. For our analysis, the inner context unit is at the organizational-level. Adapted from Aarons, et al. [9] and Aarons & Green [11]

An inductive, conventional content analysis approach was employed to examine the transcripts for leaders’ perceptions of and experiences sustaining grant-funded programs [29]. Transcripts were organized into analyzable paragraph sections, first coded a priori using the interview guide open-ended questions for topic categories (barriers, facilitator, strategies employed) [28]. These sections were then analyzed for emergent themes through open coding [30], a process by which units of text are qualified by concepts and added to a working codebook for continual reference [28]. After all sections were coded by one author, a second coded the material separately for comparison; any differences were discussed to the point of consensus [31]. Codes were then crosscut and analyzed for context and relationship to each other to solidify themes, a process known as axial coding [28, 31]. Coder agreement ranged from 68 to 92% with a median score of 81%, falling in the very good to excellent range for standard qualitative research metrics [32–34]. All analysis was completed using NVIVO 12 Pro. Once these codes were determined, they were organized within the outer and inner contexts of the EPIS sustainability framework, which reflects differential-level factors that influence adoption of evidence-based practice within a system (Fig. 1) [10]. For this analysis, the contexts include the following:

i. Outer context: For this analysis, this includes state-level systems and all public, government, and legislative entities. Subdomains include sociopolitical and systems-level dynamics such as the service environment (funding mechanisms) and inter-organizational characteristics (external leadership and public-academic partnerships) that influence adoption of a practice.
ii. Inner context: For this analysis, this pertains to the state agency within which the interviewed authority operates. Subdomains include intra-organizational characteristics (staffing and management) and organizational-level factors that facilitate adoption of a practice.

## Results

### Participant demographics

Of the 16 leaders who were interviewed, the majority (68.7%, *n* = 11) were female. The leaders most commonly led from census designated Southern states (5) followed by the West (5), Northeast (4), and Midwest (2); approximately equal to the order of census regions by population. The majority (87.5%, *n* = 14) of both leadership groups were housed in Medicaid expansion states, which adjusted eligibility to uninsured individuals at or below 138% of the federal poverty level. This is slightly over-representative of the national Medicaid expansion proportion of 74% of states [35]. Seventy-five percent (*n* = 6) of the current leaders were housed within their states’ department of health, with 50% (*n* = 4) of those within divisions of behavioral and mental health and 25% (*n* = 2) in substance use departments, exclusively. The remaining two leaders led from within departments of child welfare and human services, respectively. The current leaders had an average of 5.3 years of experience in the role of state agency authority and an average of 12.9 years in the substance use field. The former leaders spent an average of 6.7 years in the role and had a cumulative average of 20.1 years in the substance use field. Seven of the eight current leaders reported directly to an appointee of the governor or legislature and half of all leaders were also appointees themselves.

Emergent themes from the responses to the a priori questions on barriers and facilitators to sustaining federal programs are listed in alignment with the EPIS framework (Table 1).

**Table 1:** Emergent themes: barriers and facilitators categorized within EPIS framework

	Theme responses by leader type	
	Current (*n* = 8)	Former (*n* = 8)
	n (%)	n (%)
**SUSTAINMENT BARRIERS**		
**Outer Context**		
Service-Environment		
Medicaid coverage & reimbursement	4 (50.0)	4 (50.0)
Rely on continued funding	3 (37.5)	X
Infrastructure	2 (25.0)	3 (37.5)
Inter-organizational		
Reduced timeframes	4 (50.0)	X
Opioid-specific funding cliff	3 (37.5)	X
Opioid-specific data collection	2 (25.0)	X
**Inner Context**		
Intra-organizational		
Costs to integrate services	3 (37.5)	4 (50.0)
Lack of adequate workforce	2 (25.0)	3 (37.5)
Individual^†^ Adopter Characteristics		
Competing priorities	3 (37.5)	5 (62.5)
Shifting priorities	X	2 (25.0)
**SUSTAINMENT FACILITIATORS**		
**Outer Context**		
Inter-organizational		
Positioning within the state	8 (100.0)	4 (50.0)
Access to governor office	3 (37.5)	X
Access to Medicaid office	6 (75.0)	X
Professional networks	X	3 (37.5)
Consumer Support/Advocacy		
External pressure/public support	2 (25.0)	3 (37.5)
Service-Environment		
Flexibility in spending	2 (25.0)	2 (25.0)
Technical assistance	3 (37.5)	1 (12.5)
**Inner Context**		
Intra-organizational		
Strategic planning	6 (75.0)	4 (50.0)
Demonstration of outcomes	1 (12.5)	X

X: Not identified as a theme among interviewees in this group

*Note*. This table only reflects responses to the barrier and facilitators prompt, in-text citation may differ when referencing separate prompt

^†^“Individual” within the inner context of the EPIS framework is interpreted as unit-level factors influencing adoption of a practice within a system. For our analysis, the inner context unit is at the organizational-level

### Emergent themes: barriers

#### Outer context: service environment

Outer context barriers included Medicaid and reimbursement challenges for programs needing funds beyond the allocated period. Half of the current (*n* = 4) and former (n = 4) leaders identified reimbursement, both within and outside of Medicaid expansion, as one of the most prominent barriers to sustaining grant-funded programs. Former leader 2 (Medicaid expansion state) described their experience attempting to get a Screening, Brief Intervention, and Referral to Treatment (SBIRT) program “reimburs[ed] through Medicaid and Medicaid commercial insurance” and their inability to “sustain that by making it kind of [a] billable insurance service.” Some leaders acknowledged this was particularly true for differential reimbursements across formularies of addiction medication (medications for addiction treatment [MAT]). Current leader 1 (Medicaid expansion state) described an inability to “have Medicaid coverage for MAT in our state…particularly for methadone.” Current leaders (*n* = 3) pointed out that certain treatment-based programs which relied on additional funding inherently posed challenges to sustainment beyond the life of the grant. One reported:

“So, for those initiatives that are new like sober living… or recovery support services through… the discretionary grants [make] sense. But, treatment is largely funded now in this country like healthcare is funded and if we're going to look at expansions and treatment services - providers need sustainable payer resources… When the money's gone. It's gone.”

- Current leader 5, Medicaid expansion state

A major non-funding related barrier that emerged among both current (*n* = 2) and former (*n* = 3) leaders was lack of ability or allocation by the grant itself to support projects that develop infrastructure. Former leader 3 described this difficulty to “build infrastructure on funding that’s going to go away… what was most badly needed is to expand both our treatment and prevention infrastructure.” Leaders often followed this up with an example of a project that addressed an “immediate” need – such as naloxone kits (current leader 5) – in the absence of developing treatment capacity.

#### Outer context: inter-organizational

Another major theme identified by half of the current leaders (*n* = 4) interviewed was the impact of condensed grant timelines on project selection and ability to integrate sustainability planning. Current leader 8 questioned, “When do you have time to build in the sustainability” for projects with time-limits when it “potentially loses the sustainability factor if you aren’t given the time to plan.” Current leader 2 touched on how shorter implementation time can make agencies “a little more cautious and risk averse” to add in innovative programs to address infrastructure.

Current leadership recognized that the limited period of grant funding immediately impacted projects funded through the opioid response grant mechanism. Many current leaders (*n* = 3) described concerns that the impending funding cliff was at odds with the non-fleeting nature of the epidemic. For example, current leader 3 described substance use disorders as “[a] chronic illness, it’s not like this is an epidemic that’s going to end” and that without continuation of funding for “some of these services I see people returning back to illicit opioids.” One quarter of current leaders (*n* = 2) also expressed concerns about inability to create data collection infrastructures within the time limit. Current leader 4 said, “the only way I can do that is I can build something internally. This is two-year money… I’m still probably a year away from having a system to actually gather this information.”

#### Inner context: intra-organizational

There was high recognition among current (*n* = 3) and former leaders (*n* = 4) that the impending grant end would leave certain programs unfunded, a cost that would have to be subsumed and integrated with other entities in order to continue. Current leader 6 questioned - in relation to the community behavioral clinics they served - “what is going to be billable, do they have the staff resources they need to keep things going?.” This concern was often mentioned in the context of integrated care between service entities. Current leader 8 mentioned the particular need within “hospital settings… if they [could] bill … services to Medicaid and find a way to absorb the costs of some of the ancillary services.” Former leaders also highlighted the barrier of dis-integrated care. Former leader 4 highlighted the lack of joint-care prioritized in the grants themselves “as we see more and more focus on just integration in general, whether it’s integration of state agencies… integration of behavioral health with physical health… there should be this focus on integration of the funding.”

Many former (*n* = 3) and current (*n* = 2) leaders discussed lack of adequate workforce, both with the skillsets and availability, to continue projects started within grant funding. Current leader 4 described this impact on system capabilities as “the lack of workforce, that’s really what constrains the expanding [sic] provider capacity.” Another, former leader 8, introduced that even if funding may exist to continue a program, “the fact that we had so few people who were specifically qualified to treat adolescents and young adults, was a problem for other funders.” Current leader 4 also mentioned provider stigma, where “bias within the practice setting” might “alienate their other patients.” Capacity, qualifications, and stigma were all workforce components that were acknowledged by leaders as prohibitive factors in sustaining grant funded programs.

#### Inner context: individual adopter characteristics

Competing priorities between funded projects was overwhelmingly identified by both the former (*n* = 5) and current leaders (*n* = 3) as the most prominent barrier to sustaining programs; spanning time, resources, and projects. Current leader 2 discussed this strain on time and staffing, saying “there’s a competition between my time… I didn’t get extra staff to implement these grants, and they’re so fast.” Former leader 4 contextualized this as their agencies’ differential “attention [paid] to the fast block grants rather than some of the smaller ones.” Current leaders (4 and 7) noted differences that exist between state and federal agency priorities as some “organizations will get funding from SAMHSA, and create a whole new program that may not be on the state priority list” in which later on the project may “not make the cut and thing[s] will not continue.” A small number of former leaders (*n* = 2) contextualized a shift in prioritization as the substance use landscape transitions from one substance to another, creating a “moving target” of different approaches and resources:

“I would also add the moving target of problems –for example the opioid money … as the problem shifts to cocaine or methamphetamine or marijuana, whatever works for one thing doesn’t always work for another.”

- Former leader 7

### Emergent themes: facilitators

#### Outer context: inter-organizational

Across both leaders, the most commonly cited outer context facilitator was one’s political positioning within the state. All current leaders (*n* = 8) and half of the former leaders (*n* = 4) discussed how their standing within state government either contributed to, or hampered, their efforts to enact change. They noted that there was a “political reality” (current leader 5) which officials felt obliged to operate within. One, former leader 6, indicated that support “from above… makes it more likely that the work can continue” after a grant has ended.

Furthermore, respondents pointed to multiple players they felt required to negotiate and collaborate with, including Medicaid, the governor’s office, and others. As a sub-theme of positioning within the state, collaboration with the governor’s office was mentioned by some current leaders (*n* = 3). Current leader 6 pointed out the importance of the governor’s support, saying “I certainly think that having people buy in at every level… from the governor’s office, all the way down, we have an absolute buy-in about the need for this kind of work.” More noticeably however, current leaders (*n* = 6) endorsed that their relationship with the Medicaid office was an important facilitator. Those leaders who enjoyed a “good relationship” (current leader 8) were able to collaborate by virtue of state bureaucratic infrastructure. As current leader 7 noted: “we have a really strong partnership with our Medicaid agency…some of that is the fact that this is a separate state agency that’s part of the governor’s cabinet that works on addiction issues and that it’s not merged together with mental health and/or with health, generally.” These kinds of bureaucratic structures seemed to matter insofar as they also impacted the ability of officials to work seamlessly. For example,

“The further removed you are from the Office of Medicaid, the harder it is to influence Medicaid policy. So being in the same department, having the director of Medicaid as a peer… facilitates being able to integrate the benefit in meaningful ways.”

- Current leader 4

Similarly, some former leaders (*n* = 3) also cited professional networks more broadly, not necessarily solely within government, as being important outside facilitators. As one former leader reported:

“One thing that helped me accomplish one of the goals in the state ... was that I knew who to contact… So I called a colleague in public health who then figured out who I could access that was external to government [who] helped us… [So] getting people that are external – that’s the facilitator. Having a leader, having someone in the leadership team that has good connections with people outside of the state… getting expertise from outside of the industry, this was definitely something that I encouraged.”

- Former leader 7

#### Outer context: consumer support/advocacy

Current (*n* = 2) and former (*n* = 3) leaders pointed to public support and external pressure as outer facilitators as they put pressure on governments to increase resources. According to former leader 7, “getting that public support is important…particularly if what you wanted to do with that public funding… is going to require new funds to continue things moving forward.” Former leader 4 also agreed, noting that this is critical given the scope and heightened public awareness of the opioid crisis:

“We all have, I think, recognized now that the opioid epidemic has gotten everyone’s attention, which, I think we can use to our advantage as a substance use disorder treatment community. We’ve been wanting for years for others to recognize the chronic… nature of addiction… there’s a lot of pressure for providers to demonstrate quality, to demonstrate outcomes, and to position themselves within the true healthcare community, if you will.”

- Former leader 4

#### Outer context: service-environment

Some current leaders (*n* = 2) identified flexibility in spending as a facilitator. Current leader 2 noted that “traditionally funding services [are] so siloed” but – in relation to the opioid response grants - “this type of funding [opioid response grants] that is so flexible…it really does help with removing silos.” Current leaders (*n* = 3) also reported that knowledge retention, sometimes in the form of technical assistance from outside contractors, was a helpful resource.

#### Inner context: intra-organizational

Among former leaders (*n* = 4), strategic planning was the most frequently mentioned facilitator. Former leader 7 created and continually adapted a strategic plan “with the goal that within our resources, [the program] was already something we were going to maintain,” thus bringing sustainment into the conversation earlier. Another added that strategic planning involved viewing time-limited grants not as isolated initiatives, but as “pilot or demonstration projects” (former leader 9) whose results could be collected and built upon for future endeavors. Current leaders (*n* = 6) also reported that strategic planning was helpful in past sustainment efforts, although none identified planning as a facilitator of current project sustainment. Four of the six who acknowledged planning as important cited the benefits gap or needs analyses. One, current leader 2, said because of gap analysis, “we [already] knew where we wanted to go. So when the funding came… it was just like, ‘Okay, we know that we have an issue in this area, so we’re going to put funding over here’.” Preemptively gaining an understanding of the most urgent needs, pre-existing coverage, and the “policy landscape and… infrastructure,” current leader 5 added, enabled leaders to “try to build and grow and sustain and create good policy… [even] if the funding goes away.”

When asked about program sustainment supports, both former and current leaders also discussed the importance of a program’s ability to demonstrate outcomes, with current leader 6 characterizing demonstration of outcomes as “building a business case for return on investment… pushing agendas and making sure that people see… return on investment exists.” Although only one current leader explicitly identified demonstration of outcomes as a facilitator, several current leaders (*n* = 3) expressed interest in receiving training on it and many former leaders (*n* = 4) recommended discussing the topic with current leaders (not listed in Table 1). Commonly cited was the need to collect and present program data in formats understandable by state legislatures. Current leader 6, who identified this as a facilitator, said they intentionally use data showing “impact and… [returns on investment]” to catch the attention of “an interested lawmaker who looks to us for… the newest, latest, and greatest thing that needs to be supported and sustained.”

### Emergent themes: sustainment strategies

When prompted to discuss strategies taken to sustain current or former programs within their tenure, leaders often highlighted initiatives that directly addressed the aforementioned barriers, which are listed in alignment with the EPIS framework (Table 2).

**Table 2:** Emergent Themes: Strategies categorized within EPIS framework

	Theme responses by leader type	
	Current (*n* = 8)	Former (*n* = 8)
	n (%)	n (%)
**Outer Context**		
Inter-organizational		
Involving stakeholders	6 (75.0)	X
Technical assistance	3 (37.5)	X
Creating buy-in	2 (25.0)	X
Selecting champions	1 (12.5)	X
Engaging leadership	3 (37.5)	8 (100.0)
Enacting policy change	1 (12.5)	2 (25.0)
Consumer Support/Advocacy		
Raising awareness	4 (50.0)	X
**Inner Context**		
Intra-organizational		
Workforce/credentialing	5 (62.5)	3 (37.5)
Capacity development	6 (75.0)	1 (12.5)
Demonstrating outcomes	2 (25.0)	X
Primary prevention initiatives	2 (25.0)	X

X: Not identified as a theme among interviewees in this group

#### Outer context: service environment

Financing was a major strategy employed among the current leaders (n = 5) and ranged from “working with our legislature to get them to approve funding for putting methadone on the formulary for Medicaid” (current leader 1) to “[writing] a new 1115 waiver that included … behavioral health advancements” (current leader 2) to adding “Medicaid as a reimbursement structure for peer support” (current leader 4). Current leader 2 specifically mentioned financial reimbursement impact on sustaining programs because “with some of the other short-term funding … the sustainability is really impacted because you’re not going to do projects necessarily increasing access.” Although not a stated strategy, there was also a high recognition among current leaders (*n* = 3) of wanting to receive training or content knowledge on healthcare financing, a separate question.

#### Outer context: inter-organizational

A major strategy employed by the current leaders (*n* = 6) included involving stakeholders early on in discussions and initiatives to create ownership for sustaining programs. For many who noted this, it included technical assistance activities (*n* = 3), creating buy-in (*n* = 2), and selecting champions (*n* = 1). Current leader 2 described the importance of creating this buy-in by “talking about sustainability … as we’re reaching across silos of different systems – how do we get them to understand and own the problem, too?”

A related strategy strongly employed by both current (n = 3) and all former (*n* = 8) leaders was engaging other state department leaders and offices and using one’s position to partner with and advocate for policy and funding changes. Former leader 3 described this inter-agency connectivity “…as a Cabinet-level official, I was able to work with the Physician General, and we called health insurers all over the state, and raised a half a million dollars for naloxone for police.” Another described this role positioning as a key component of change as “the biggest thing … where they [the leader] are in the hierarchy and where their sphere of influence actually is” (former leader 7).

Enacting policy changes was another avenue employed by leaders [former (*n* = 2), current (*n* = 1)]. Former leader 7 described their plan to change licensing policies as “one of the plans to sustain was to change the contract language or changing … the rules and regulations.” Another, former leader 5, explicitly described rewriting “bundle payments differently for OTPs… so even though this money will go away, their rate for all other state and federal money will support the program.” Another strategy acknowledged by current leaders (*n* = 4) was raising awareness through information campaigns –categorized within the “consumer support/advocacy” subdomain of the outer context (Table 2).

#### Inner context: intra-organizational

Several strategies were stated by former (*n* = 3) and current leaders (*n* = 5), respectively, to address barriers related to workforce capacity. This included prioritizing “co-location models” (current leader 8) to “hiring people in government [with] business experiences,” because “learning about insurance and about how the healthcare world works … never used to be so essential as it is now” (former leader 6). Former leader 8 even discussed the intentionality of workforce capacity as a key component of sustainability, providing an example of “credentialing over 300 folks to be peer specialists … purposefully put into both the [opioid response] grants to encourage that sustainability.”

The majority of current leaders (*n* = 6) and one former leader mentioned employment of capacity development initiatives to build content knowledge among practitioners, lasting beyond the grant-funding period. This included training initiatives on diversion and medication-assisted therapy within jail settings (current leader 2), hospital training collaboratives, and telehealth technology among primary care providers (current leader 4). Current leader 8 contextualized using these grant funds as an opportunity to “to be paying for activities that put things in place like … a good knowledge basis and some training … that they can build from any kind of workforce development.”

Other minor strategies listed by current leaders included the importance of demonstrating outcomes (*n* = 2) and engaging in primary prevention initiatives (n = 2).

## Conclusions

### Main findings

States received unprecedented funding through the recent twenty-first Century Cures Act opioid response legislation. Our findings have particular relevance to the current grants and document levers to sustain their impact beyond their allotted funding timeline. The analysis revealed financing and reimbursement, service integration, and workforce capacity as the most commonly cited barriers to sustainment. Status in state government structure, public support, and spending flexibility were noted as key facilitators. The most commonly cited barriers – “costs and integration of services” and “Medicaid coverage and reimbursement” – aligned with the intra-organizational domain of the EPIS inner context and the service-environment funding domain of the outer context, respectively (Table 1). The most commonly cited facilitators – “positioning within state government” and “external pressure/public support” – fell into the inter-organizational domain of the EPIS outer context. Initiatives to help states sustain programs may benefit from orientation to outer context components (leadership, policies, funding, and collaborations), given their prevalence as stated barriers and facilitators [8].

### Recommendations and alignment

Here were present the following recommendations based on alignment with the literature and results. We recommend a strong orientation of strategies targeted at the outer context to equip leaders with strategies to navigate systems they work within.

#### Development of leadership capacity

Several themes were salient across the identified barriers, facilitators, and sustainment strategies. The most highly cited strategy for sustainment, “engaging leadership,” might be interpreted as the actionable version of the most commonly cited outer sociopolitical facilitator theme, “positioning within the state” (Table 2). This may point to the political nature of the leaders’ roles, and the applicability of sustainment strategies targeting sociopolitical dynamics. It also points to a need to develop leadership capacity in support of implementation through networking, collaboration, and engagement of stakeholders – another stated strategy.

#### Framing and messaging training

Other key facilitators identified among leaders were “external pressure/public support” and “raising awareness,” which stated as a strategy reiterate both the high visibility and urgency of the opioid response landscape. Training in messaging, framing, and marketing may benefit leaders whose continued projects depends on the public awareness, perception, and aligned political will across state divisions.

#### Economic and financial modeling expertise sharing

Financing was often identified as a barrier to sustainment for both former and current leaders, but only cited as a common strategy among current leaders. This might indicate an increased priority placed on financing in the post-expansion landscape, but leaders may still lack the expertise to navigate this burgeoning field. Here, strong academic partnerships may play a key role in delivery of economic data and best-practices for integrating financial models into state planning.

Overall, while leaders noted their advancements and an obligation to sustainment, they consistently reiterated their lack of resources to execute them effectively. A commitment to sustainment is of little value if leadership lacks the tools to make it happen. In understanding these barriers to better target sustainment strategies [25], policy practitioners can create targeted supports and tools for states to increase longevity of substance use treatment programs.

#### Literature alignment

These findings align with a recent literature review on implementation determinants in the policy realm [36]. Here, organizational climate, actor relationships within networks, and public awareness and knowledge – all outer context components – were the most highly cited determinants of successful policy [36]. It aligns with one study recognizing outer setting contexts as a driver of evidence-based practice uptake within the adoption phase of implementation [37]. Many of the themes identified in the interviews align with quantitative studies which identified political support, funding stability, partnerships, and strategic planning as important domains for sustainability, generally [8, 38]. Themes in this study heavily aligned with the outer context, similar to the emerging literature on policy determinants [8, 36, 37]. However, a recent systematic review of the EPIS framework found it makes up a lower proportion of implementation research overall [20].

The methods in this study and corresponding results answer a call for more exploratory qualitative and mixed-methods research on factors affecting sustainment [11, 37, 39, 40]. It is the first to qualitatively identify barriers and facilitators among policy-facing leaders within the sustainment phase and in-relation to opioid epidemic funding [6, 11]. It is also among the first to present sustainment strategies in alignment with a stated sustainability framework [6, 11].

### Limitations and next steps

This study has several key limitations - notably the small, nonrandom sample of state authority leaders. While our study represents a significant percentage of individuals who hold these roles, this limits the ability to generalize results to leaders in different government roles or health service fields. The study encountered common restrictions of qualitative work, i.e. the difficulty of measuring contextual determinants (ex: specific strategies employed by states) while preserving participant confidentiality. Although we allowed themes to emerge inductively, they were grouped by a priori prompts of barriers, facilitators, and strategies. The responses elicited by the interviewees may therefore be limited to the questions that were asked, though standardized across the study. Additionally, the nature of qualitative content analysis does not allow causal inferences from the data, but can inform initial and iterative framework and strategy development [41].

Continued research must focus on improved understanding of organizational and political drivers of sustainment [4, 5, 20, 42]. The field of addiction policy needs sustainment strategies developed from an understanding of key drivers [24, 40, 42, 43]. The present analysis provides insight into effective sustainment strategies deployed by policy-facing state leaders to extend time-limited programs. It adds to the growing literature supporting identification of barriers and facilitators in implementation, which must be strategically linked to sustainment ventures [24–26]. Utilizing these findings may help in the development of feasible, tailored, and effective strategies that state health care authorities can use [4, 24, 25]. Content training and expertise-sharing through academic partnerships may equip leaders with tools to employ strategies aimed at specific barriers. As state systems leaders work with temporarily extended twenty-first Century Cures Act funding, it is important to make sustainment a priority. Failure to plan how to sustain effective programs for persons with opioid use disorders will cost lives.

## Data Availability

The transcripts generated and/or analyzed during the current study are not publicly available due to interviewee confidentiality. Though anonymized, information provided by participants may make them identifiable given the small sample pool. However, they may be made available from the corresponding author on reasonable request.

## Abbreviations

SAMHSA: Substance Abuse and Mental Health Services Administration
FDA: U.S. Food and Drug Administration
EPIS: Exploration preparation active implementation and sustainment framework
SSA: Single State Authority
MAT: Medications for addiction treatment
OTPs: Opioid treatment programs
SBIRT: Screening, Brief Intervention, and Referral to Treatment
